# Stimulation of regulatory T cells with *Lactococcus lactis* expressing enterotoxigenic *E. coli* colonization factor antigen 1 retains salivary flow in a genetic model of Sjögren’s syndrome

**DOI:** 10.1186/s13075-021-02475-1

**Published:** 2021-04-06

**Authors:** Ali Akgul, Massimo Maddaloni, Sang Mu Jun, Andrew S. Nelson, Vanessa Aguilera Odreman, Carol Hoffman, Ella Bhagyaraj, Alexandria Voigt, Jeffrey R. Abbott, Cuong Q. Nguyen, David W. Pascual

**Affiliations:** 1 Department of Infectious Diseases & Immunology, College of Veterinary Medicine, University of Florida, P.O. Box 110880, Gainesville, FL 32611 United States; 2 Department of Veterinary Microbiology & Pathology, Washington State University, P.O. Box 647040, Pullman, WA 99164 United States

**Keywords:** Sjogren’s syndrome, Regulatory T cells, *Lactococcus lactis*, Colonization factor, Antigen 1, IL-10, TGF-β

## Abstract

**Background:**

Sjögren’s syndrome (SjS), one of the most common autoimmune diseases, impacts millions of people annually. SjS results from autoimmune attack on exocrine (salivary and lacrimal) glands, and women are nine times more likely to be affected than men. To date, no vaccine or therapeutic exists to treat SjS, and patients must rely on lifelong therapies to alleviate symptoms.

**Methods:**

Oral treatment with the adhesin from enterotoxigenic *Escherichia coli* colonization factor antigen I (CFA/I) fimbriae protects against several autoimmune diseases in an antigen (Ag)-independent manner. *Lactococcus lactis*, which was recently adapted to express CFA/I fimbriae (LL-CFA/I), effectively suppresses inflammation by the induction of infectious tolerance via Ag-specific regulatory T cells (Tregs), that produce IL-10 and TGF-β. To test the hypothesis that CFA/I fimbriae can offset the development of inflammatory T cells via Treg induction, oral treatments with LL-CFA/I were performed on the spontaneous, genetically defined model for SjS, C57BL/6.NOD-*Aec1Aec2* mice to maintain salivary flow.

**Results:**

Six-week (wk)-old C57BL/6.NOD-*Aec1Aec2* mice were orally dosed with LL-CFA/I and treated every 3 wks; control groups were given *L. lactis* vector or PBS. LL-CFA/I-treated mice retained salivary flow up to 28 wks of age and showed significantly reduced incidence of inflammatory infiltration into the submandibular and lacrimal glands relative to PBS-treated mice. A significant increase in Foxp3^+^ and IL-10- and TGF-β-producing Tregs was observed. Moreover, LL-CFA/I significantly reduced the expression of proinflammatory cytokines, IL-6, IL-17, GM-CSF, and IFN-γ. Adoptive transfer of CD4^+^ T cells from LL-CFA/I-treated, not LL vector-treated mice, restored salivary flow in diseased SjS mice.

**Conclusion:**

These data demonstrate that oral LL-CFA/I reduce or halts SjS progression, and these studies will provide the basis for future testing in SjS patients.

**Supplementary Information:**

The online version contains supplementary material available at 10.1186/s13075-021-02475-1.

## Background

Sjögren’s syndrome (SjS) is an autoimmune chronic inflammatory disease affecting primarily the lacrimal and salivary glands. SjS presents as dry mouth and dry eyes as a consequence of diminished salivary and lacrimal flow [1]. SjS is classified as either primary Sjögren’s syndrome or secondary Sjögren’s Syndrome; the latter being the result of other autoimmune disorders, including systemic lupus erythematosus (SLE), rheumatoid arthritis (RA), and systemic sclerosis. SjS is a debilitating disease affecting 3.1 million individuals in the USA [2], with woman being nine times more likely to be afflicted than men [2, 3]. In addition to secretory dysfunction, SjS can also have systematic manifestations affecting the skin, gastrointestinal tract, lungs, blood vessels, liver, pancreas, kidneys, and peripheral and central nervous systems [1, 4]. SjS is characterized by lymphocyte infiltrates in the glands. Both T cells and accompanied activated B cells have pathogenic roles in primary Sjögren’s syndrome immunopathology [5, 6].

Several mouse models have been used to recapitulate aspects of SjS. NOD mice develop salivary and lacrimal secretory dysfunction, which begins with the loss of secretory function by 20 wks of age [7–9]. An  early study identified that Idd3 and Idd5 loci are required for secretory gland dysfunction in NOD mice [9]. When both NOD-derived regions were introduced into C57BL/6 mice, the C57BL/6.NOD-*Aec1Aec2* strain was created [10]. This mouse strain produces SjS-like disease, displaying pathophysiological changes at early age, followed by lymphocytic infiltrations of the salivary and lacrimal glands, and the production of autoantibodies (autoAbs) to nuclear antigens (Ags; SSA/Ro, SSB/La) in the complete absence of type 1 diabetes. The presence of inflammatory infiltrates [11, 12] coincided with increased proinflammatory cytokine production of IL-17, IL-22, and IL-23. Another SjS murine model is the MRL/lpr mouse, which was developed with a genetic mutation of the lymphoproliferation (*lpr*) gene on chromosome 19 [13], and it spontaneously develops disease similar to SLE [14] and RA [15] characterized by splenomegaly, arthritis, glomerulonephritis, and massive lymphadenopathy [16]. These mice develop a SjS-like phenotype, but only 30% of mice develop anti-SSA/Ro and anti-SSB/La Abs [17]. In contrast, C57BL/6.NOD-*Aec1Ace2* mice exhibit a quicker disease onset than NOD mice in terms of both SjS-like pathophysiology and secretory dysfunction [18]. As with MRL/lpr or NOD mice, C57BL/6.NOD-*Aec1Ace2* mice produce elevated levels of autoAbs. AutoAbs produced by these autoreactive B cells are directly linked to SjS secretory dysfunction [19].

Human and mouse studies revealed the increased interferon (IFN)-γ and IL-17A cytokine presence in salivary glands and plasma, which contribute to the pathogenesis of SjS [19–22]. Earlier studies in mice revealed that IFN-γ plays an early role in disease development attracting invasive lymphocytes and hampering gland development. Similar to murine studies, SjS patients exhibit high levels of IFN-γ and IFN-responsive factors. The upregulation of the IFN pathway induces the activation of macrophages, natural killer (NK) cells, and CD8^+^ T cells. Pathogenic T helper Th (17) cells also significantly contribute to disease development [11, 19, 20, 23–25].

Currently, replacement therapies such as artificial saliva and eye lubricants and immunosuppressive agents are used to treat SjS patients [26, 27]. B cell-directed therapies, e.g., rituximab (anti-CD20 monoclonal Ab [mAb]) [27–29], and immune gene therapy using IL-27 to suppress Th17 cells can result in broad immunosuppression, making patients more susceptible to infections [28–31]. However, these interventions do not address the cause of SjS, and none redirects T cell responses to dampen the inflammatory response.

Oral treatment with colonization factor antigen I (CFA/I) fimbriae from enterotoxigenic *Escherichia coli* can prevent and treat experimental autoimmune diseases for experimental autoimmune encephalomyelitis (EAE), arthritis, and type 1 diabetes [32–35]. The strength of CFA/I fimbriae therapeutic effect is its ability to elicit regulatory T cells (Tregs) via the production of regulatory cytokines IL-10, IL-13, IL-35, and TGF-β [36, 37]. CFA/I fimbriae stimulate Tregs in a bystander fashion to impact auto-Ag-specific Tregs as well [32–37]. In the arthritis-related studies, CFA/I fimbriae elicited a heterogeneous population of CD39^+^ Tregs, where a portion were IL-10^+^ Foxp3^+^ and another portion being TGF-β^+^ Foxp3^−^ [36, 37]. Not limited to a single population, CFA/I fimbriae elicited both CD25^+^ CD4^+^ and CD25^−^ CD4^+^ Tregs, each capable of providing protection against EAE [32]. Thus, the advantage of using CFA/I fimbriae is that it enables treatment of autoimmune diseases without prior knowledge of the auto-Ag, and the types and degree of heterogeneity of the Tregs elicited are dependent on the disease. Probiotics have also been used to implement immune homeostasis to reduce autoimmunity [38–40].

Historically, lactic acid bacteria (LAB) represent the core of probiotic-based interventions, although some nonpathogenic *E. coli* [41–43] attenuated *Salmonella* [44], *Bifidobacterium spp*. [45], and some yeasts, e.g., *Saccharomyces boulardii* [46], and also proved to be valuable tools as novel therapeutic and prophylactic interventions. Amongst these, LABs are considered ideal vectors for oral or mucosal delivery since they are inherently nonpathogenic, and they can survive the harsh conditions of the gastric environment. LABs are amenable to recombinant expression of passenger Ags to stimulate immunity against a number of pathogens [47–49], to curb the effects of inflammatory diseases [50–53], and to reduce proliferation of cancer. Given its therapeutic impact, we queried whether our *Lactococcus lactis* expressing CFA/I fimbriae (LL-CFA/I) can be used to treat SjS-like disease in the C57BL/6.NOD-*Aec1Aec2* genetic model.

## Methods

### Mice

The genetically defined C57BL/6.NOD-*Aec1Aec* (SjS) female mice used to mimic human SjS were bred locally. C57BL/6 (B6) females were obtained from Charles River Laboratory (Frederick, MD, USA). All mice were housed under specific pathogen-free conditions and provided with food and water ad libitum. Mice were allowed to acclimate to the facility for at least 5 days prior to handling. All animal experiments in these described studies were conducted in strict accordance with the recommendations in the Guide for the Care and Use of Laboratory Animals of the National Institutes of Health. All animal procedures were approved by the University of Florida Institutional Animal Care and Use Committee.

### *Lactococcus* culture conditions

*Lactococcus lactis* IL1403 carried the pMSP3535H3 as an empty state (vector control) or bearing the *E. coli cfaI* operon as described previously [34]. The pMSP3535H3 [54] was a kind gift provided by Dr. David Mills from the University of California—Davis. Briefly, starter small cultures were grown overnight 30 °C, and next day, large culture induced with 0.5 μg/mL nisin (Sigma-Aldrich, St. Louis, MO). Four hours after induction, SjS mice were orally gavaged with sterile 10% sodium bicarbonate solution to neutralize stomach acid. After 5 min, mice were orally gavaged with the specified doses (5 × 10^7^ to 5 × 10^9^ CFUs) of LL-CFA/I or LL vector in sterile PBS; negative control mice received only sterile PBS. Additional LL growth conditions are provided in Additional File 1.

### Measurement of salivary flow rate (SFR)

To measure stimulated flow rates of the saliva, individual mice were weighed and given an intraperitoneal (IP) injection of 100 μl of a mixture containing isoproterenol (Sigma-Aldrich) (0.2 mg/1 ml of PBS) and pilocarpine hydrochloride (Sigma-Aldrich) (0.05 mg/1 ml in PBS). Saliva was collected for 10 min from the oral cavity of individual mice using a micropipette as previously described [25]. The volume of each saliva sample was measured to calculate salivary flow rates.

### Histology

To measure extent of inflammation, the salivary glands were fixed in 10% phosphate-buffered formalin (Leica Biosystems, Richmond, IL) for 24 h. Fixed tissues were embedded in paraffin and sectioned at a thickness of 5 μm. Paraffin-embedded sections were deparaffinized by immersing in xylene, followed by dehydration in ethanol, and tissue sections were stained with hematoxylin and eosin (H&E) dye (UF College of Veterinary Medicine Histology Tech Services, Gainesville, FL). To measure extent of leukocyte infiltrations in the salivary glands, a single histological section per gland per mouse was scanned using an Aperio ScanScope (Aperio, San Diego, CA) slide digitizer at ×20 magnification. Infiltrated regions were identified and calculated using the Aperio ImageScope software. The extent of the infiltrate varied between 1 mm^2^ and in 4mm^2^, and samples from each mouse for each treatment group were calculated.

### Detection of serum antinuclear antibodies (ANA)

Individual serum from each treatment group was evaluated for the presence of anti-nuclear antibodies using HEp-2 ANA kit (Inova Diagnostics, Inc., San Diego, CA, USA) as previously described [28–31]. Manufacturer’s specific directions were followed in all procedures. Each serum sample was diluted 1:80 and incubated on HEp-2-fixed substrate slides for 1 h at room temperature in a humidified chamber. After three 5-min washes with PBS, the substrate slides were treated with a 1:100 dilution of Alexa 488 goat anti-mouse IgG (H + L) (Life Technologies) for 45 min at room temperature. After three washes, Vectashield DAPI mounting medium (Vector Laboratories, Burlingame, CA, USA) was applied, and overlaid with a glass coverslip. Fluorescence was detected by fluorescence microscopy at ×400 magnification by using a Nikon microscope, and all images were obtained with exposure of 200 ms.

### Lymphocyte cell culture

Single cell suspensions from aseptically removed the head and neck lymph nodes (HNLNs), mesenteric LNs (MLNs), and spleens were prepared as previously described [35]. Lymphocytes were cultured in a complete medium: RPMI 1640 with 2 mM l-glutamine (Genesee Scientific, El Cajon, CA) containing 10% fetal bovine serum (Atlanta Biologicals, Oakwood, Georgia), plus supplements (Invitrogen, Carlsbad, CA), 100 U/mL penicillin, 100 μg/mL streptomycin, 1 mM sodium pyruvate, and 0.1 mM nonessential amino acids. Lymphocytes were cultured at 10^6^ cells/well in 96-well, round-bottomed tissue culture plates (Millipore, Billerica, MA) coated with 5 μg/mL anti-CD3 mAb (clone 17A2; Invitrogen, Carlsbad, CA, USA) plus 2.5 μg/mL of soluble anti-CD28 mAb (clone 37.51; Invitrogen) was stimulated for 48 h at 37 °C. Lymphocytes were stimulated in triplicate for 2 days for flow cytometry analysis or for 4 days for collection of cell culture supernatants, which were then stored at − 20 °C until assayed by cytokine-specific ELISAs.

### Real-time polymerase chain reaction

Total RNA was extracted from MLNs using a kit (TRIzol® Plus RNA Purification Kit, Life Technologies, New York, NY, USA) and RNeasy® (QIAGEN Inc., Valencia, CA, USA) mini kit accordance with the manufacturer’s instructions. The quality and quantity of RNA were determined by measuring the absorbance at 260 and 280 nm using NanoDrop ND-1000 UV-Vis spectrophotometer (Thermo Fisher Scientific, Waltham, MA, USA). All samples absorption ratio (A260/A280) ranged between 2.0 and 2.2. Contaminating DNA was eliminated by DNase I treatment with RNase-Free DNase Set (Qiagen). First-strand cDNA was produced by using Maxima First-Strand cDNA Synthesis Kit for RT-qPCR (Thermo Scientific).

The PCR was set to initial denaturation at 95 °C for 3 min, 42 cycles of denaturation at 95 °C for 15 s, annealing at 62 °C for 30 s, and extension at 72 °C for 30 s, and a final extension at 72 °C for 3 min. All samples were tested in triplicate. Differences in the threshold cycle (ΔCt) number were determined between the target genes and the housekeeping genes. The relative induction of mRNA expression was determined after normalization using beta actin as the reference gene, and the results are shown as relative values of mRNA expression vs. that of the control-PBS. Primers were designed from nucleotide sequences identified using NCBI BLAST (http://blast.ncbi.nlm.nih.gov/Blast.cgi) to confirm the specificity of the primer design. The primer characteristics of selected genes are listed in Table 1.

**Table 1:** Primers used for gene expression analysis

Gene name	Primer	Sequence (5`➔3`)	Amplicon size	Source
*Foxp3*	F R	AGCCATGATCAGCCTCCCAC GGGTAGGATCCTTGGGGTG	136	In this study
*Tgfβ*	F R	CTTCAATACGTCAGACATTCGG GTAACGCCAGGAATTGTTGCTA	142	[55]
*Il10*	F R	GCTCTTACTGACTGGCATGAG CGCAGCTCTAGGAGCATGTG	105	[56]
*Rorγt*	F R	GACCACACCTCACAAATTGA AGTAGGCCACATTACACTGCT	166	In this study
*Il17*	F R	GACTCCTGGGAAGACCTCATT GAGGACCTTTTGGGATTGGTA	189	In this study
*Tbet*	F R	AGCAAGGACGGCGAATGTT GTGGACATATAAGCGGTTCC	175	[57]
*Ifnγ*	F R	ATGAACGCTACACACTGCATC CCATCCTTTTGCCAGTTCCTC	152	[58]
*Tnfα*	F R	CCCTCACACTCAGATCATCTTCT GCTACGACGTGGGCTACAG	228	[56]
*βactin*	F R	ATCTACGAGGGCTATGCTCTCC AGCCTCGGTCAGGATCTTCAT	96	[59]

### Flow cytometry

Prior to staining-stimulated lymphocytes for flow cytometry analysis, restimulated lymphocytes were treated with 5 μg/mL brefeldin A (Biovision, San Francisco, CA, USA) for 4 h to block cytokine release. Splenic and LN lymphocytes were subjected to a viability stain using a LIVE/DEAD Fixable Blue Dead Cell Stain Kit, for UV excitation (ThermoFisher). Cells were then washed with Dulbecco’s PBS (Gibco, ThermoFisher**)** plus 10% fetal bovine serum (Atlanta Biologicals) and labeled with mAbs specific for TCR-β, CD4, CD8α, CD19, CD25, TGF-β (BioLegend, San Diego, CA), and CD39 (eBioscience, San Diego, CA). Cells were then fixed and permeabilized using the True-Nuclear Transcription Factor Buffer Set (BioLegend) and labeled with mAbs specific for IFN-γ, IL-17 (BD Pharmingen, San Jose, CA), GM-CSF (eBioscience), IL-10, and Foxp3 (Invitrogen). Fluorescence was acquired on a Fortessa flow cytometer (Becton Dickinson Franklin Lakes, NJ), using FACSDiva software (Becton Dickinson). All samples were analyzed using FlowJo software (BD Biosciences, Ashland, OR).

### Adoptive transfer of CD4^+^ T cells

To assess source of Tregs, lymphocytes were positively or negatively sorted. For positive sorting, pooled lymphocytes from the spleens and LNs were stained with anti-CD4 mAb (clone RM4–5; eBioscience) and sorted using a Sony H800 cell-sorter (Sony Biotechnology, San Jose, CA). For negative selection cell-sorting, magnetic bead separation was done using Dynabeads™ Untouched™ Mouse CD4 Cells (Invitrogen, Thermo Fisher). The positively or negatively sorted CD4^+^ T cells achieved ≥95% purity.

### Cytokine ELISA

At termination of the studies usually at 28 wks, the spleens, HNLNs, and MLNs were isolated and cultured as described above. Collected supernatants were stored at − 20 °C. Cytokine capture ELISAs were used to quantify levels of IFN-γ, GM-CSF, IL-6, IL-10, IL-17, and TGF-β present in the supernatants, and these were performed similar to those previously described [60, 61]. Briefly, wells were coated with purified anti-mouse mAbs: anti-IFN-γ (clone R4-6A2, 10 μg/mL; ThermoFisher), anti-GM-CSF (clone MPI-22E9, 0.5 mg/ml; Invitrogen), anti-IL-6 (clone R4-6A2, 5 μg/mL; Peprotech, Rocky Hill, NJ, USA), anti-IL-10 (clone JES5-2A5, 2 μg/mL; eBioscience), anti-IL-17 (clone TC11-18H10, 2 μg/mL; BD Pharmingen), and anti-TGF-β (clone A75-22 μg/mL; eBioscience) mAbs. For detection, biotinylated anti-mouse IFN-γ (clone XMG1.2, 0.5 μg/mL; BD Pharmingen), anti-GM-CSF (clone BVD2-21C11, 0.5 μg/mL; BD Pharmingen), anti-IL-6 (clone MP5-32C11 0.5 μg/mL; BD Pharmingen), anti-IL-10 (clone SCX-1, 1.5 μg/mL; BD Pharmingen), anti-IL-17 (clone TC11-8H4, 1.5 μg/mL; BD Pharmingen), and anti-TGF-β (clone A75-3, 5 μg/mL; BD Pharmingen) mAbs were used. The third step antibody involved a horseradish peroxidase (HRP)-conjugated goat anti-biotin Ab (Vector Laboratories), and after washing, ABTS peroxidase substrate (Moss, Inc., Pasadena, ME, USA) was added to each well. Cytokine concentrations were extrapolated from standard curves generated by recombinant murine cytokines IFN-γ (Peprotech), IL-6 (BD Pharmingen, Franklin Lakes, NJ), IL-10, IL-17, GM-CSF (eBioscience, San Diego, CA), and TGF-β (R&D Systems, Minneapolis, MN, USA).

### Statistics

A power analysis was conducted and found that 5 mice per group were needed to show a significant difference for at least a 20% change in SFR. All presented data are the mean ± standard error of the mean (SEM). Statistical significance was tested using GraphPad Prism 8 (Prism, Irvine, CA). One-way ANOVA with Tukey’s multiple comparisons test were used to compare FACS data, cell counts, cytokine production, and salivary flow rates. All results are discerned to the 95% confidence interval.

## Results

### Evaluation of SjS mice regulatory T cell (Treg) expression levels

**Fig. 1:**
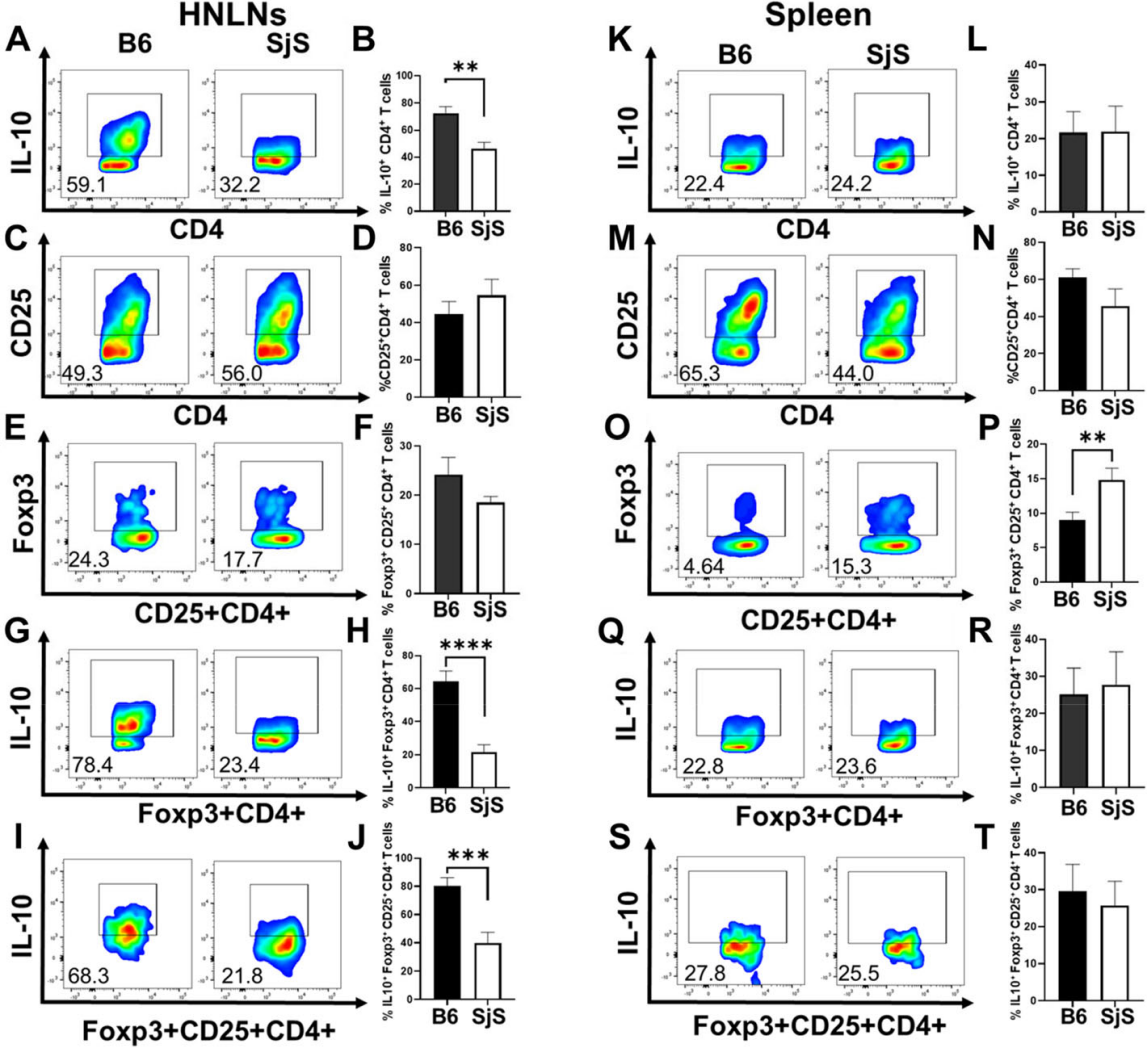
Expression of regulatory T cell (Treg) levels in C57BL/6.NOD-*Aec1Aec* (SjS) mice compared to C57BL/6 (B6) females. CD4^+^ T cell analysis was performed on two groups (*n* = 5 mice/group) of age-matched B6 and SjS females (32 weeks of age) and evaluated for the expression of IL-10, CD25, and Foxp3. Isolated lymphocytes from (**a**–**j**) head and neck lymph nodes (HNLNs) and (**k**–**t**) spleens were stained for expression of (**a**, **b**, **g**–**l**, **q**–**t**) IL-10, (**c**–**f**, **i**, **j**, **m**–**p**, **s**, **t**) CD25, and (**e**–**j**, **o**–**t**) Foxp3; **P < 0.01, ***P < 0.001, ****P < 0.0001 denote significant differences between SjS and B6 mice

CD4^+^ T cell analysis was performed on diseased SjS mice to discern if these showed a depression in Treg levels. Lymphoid tissues from groups of age-matched C57BL/6 and SjS females (32 weeks of age) were evaluated for indicators of Treg expression (Fig. 1). Isolated lymphocytes from head and neck lymph nodes (HNLNs) and spleens were stained for expression of IL-10, CD25, and Foxp3. The percentage of IL-10^+^ CD4^+^ T cells was significantly reduced (*P* < 0.05) in SjS HNLNs relative to B6 mice (Fig. 1a, b). No significant differences in the frequency of CD25^+^ CD4^+^ and Foxp3^+^ CD25^+^ CD4^+^ T cells were observed in HNLNs (Fig. 1c–f). Only the SjS spleens showed a significant (*P* < 0.01) increase in Foxp3^+^ CD25^+^ CD4^+^ T cells (Fig. 1o, p). However, IL-10-producing Foxp3^+^ CD4^+^ (Fig. 1g, h) and Foxp3^+^ CD25^+^ CD4^+^ T cells (Fig. 1i, j) were significantly (*P* < 0.001) diminished in the HNLNs, but not in the spleen (Fig. 1 k, l, q, t). No significant differences in total lymphocyte numbers for either the HNLNs or spleen (data not shown) were found, suggesting that Treg function, rather than number, may be impaired in SjS mice.

### Oral treatment with LL-CFA/I ameliorates disease in SjS mice

**Fig. 2:**
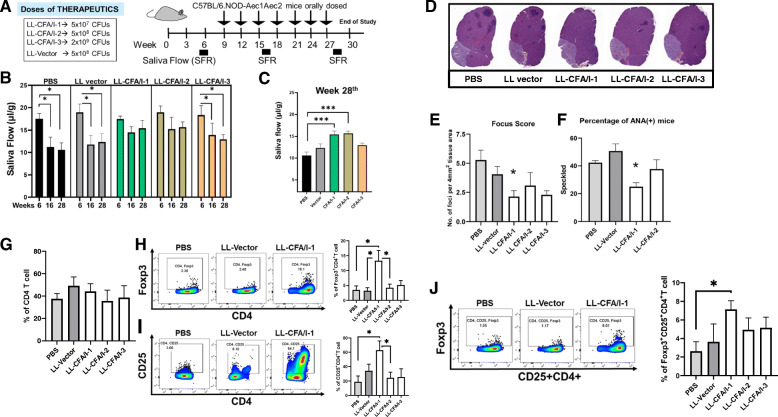
Oral treatment with colonization factor antigen 1 (CFA/I) fimbriae protects against the development of SjS. **a** Groups of 6-week-old SjS (5–8 mice/group) females were orally dosed with 5 × 10^7^ (− 1 or low dose), 5 × 10^8^ (− 2 or medium dose), or 5 × 10^9^ CFUs (− 3 or high dose) of *Lactococcus lactis*-CFA/I (LL-CFA/I), 5 × 10^8^ CFUs (medium dose) *L. lactis* (LL vector), or phosphate-buffered saline (PBS). Bacteria were grown in synthetic M17 medium supplemented with 0.5% glucose. Additional doses were administered to the mice every 3 weeks. **b** Saliva flow rate (SFR) measurements were taken prior to treatment, and 16 and 28 wks of age; *P < 0.05 versus 6-wk measurement. **c** Analysis at 28 wks of age show that low and medium doses of LL-CFA/I prevent reductions in SFR compared to PBS-treated mice; ***P < 0.001, one-way ANOVA followed by Dunnett’s multiple comparisons test was performed. **d** At 28 wks of age, submandibular glands were formalin fixed and stained with hematoxylin and eosin to determine extent of inflammatory cell infiltration. Representative images of stained tissues at ×20 magnification. **e** Infiltrated regions were drawn for area determinations and calculated by using the Aperio ImageScope software. Focus score of infiltrates were determined by using average focus size in area of foci in 4 mm^2^; *P < 0.05 versus PBS-treated mice. One-way ANOVA followed by Dunnett’s multiple comparisons test was performed. **f** Antinuclear antibodies (ANA)-positive mice from LL-CFA/I- and vector-treated mice compared to PBS-treated group; *P < 0.05. **g**–**j** For each sample, flow cytometry analysis for regulatory T cells (Tregs) was performed on 1 × 10^6^ lymphocytes isolated from mesenteric LNs (MLNs) after 1 week following the last treatment dose. **g** Percentages of CD4^+^ T cells were not significantly different amongst the treatment groups. The percentages of **h** Foxp3^+^CD4^+^, **i** CD25^+^CD4^+^, and **j** Foxp3^+^CD25^+^CD4^+^ T cells are depicted; *P < 0.05 relative to PBS- or LL vector-dosed mice

To evaluate LL-CFA/I’s efficacy to prevent disease in SjS mice, groups of 6 week (wk)-old females were tested for their individual saliva flow rate (SFR) [62] showing a mean baseline rate of 18.29 ± 0.31(Fig. 2b). Mice were subsequently orally dosed with PBS, LL vector, or LL-CFA/I. Three doses of LL-CFA/I were tested: a low dose of 5 × 10^7^ CFUs, a medium dose of 5 × 10^8^ CFUs, and a high dose of 5 × 10^9^ CFUs. Additional doses were administered at 3-wk intervals (Fig. 2a). Untreated SjS mice showed a time-dependent progressive reduction in their SFR [62] as evidenced by the group receiving PBS only. The low- and medium-dose-treated LL-CFA/I groups were significantly protected against SFR loss (*P* < 0.001) when measured at 16 and 28 wks of age (Fig. 2b). At termination of the study, SjS mice treated with the high dose of LL-CFA/I failed to maintain salivary flow when compared to mice administered lower treatment doses (Fig. 2c). The reduction in SFR by LL vector-treated SjS mice did not significantly differ from the PBS-treated group (Fig. 2b, c). Hence, CFA/I fimbriae conferred protection against SFR loss, and the protection is dose-dependent.

### Low-dose LL-CFA/I treatment reduces inflammation of salivary glands, lessens autoAb generation, and increases Tregs

By 28 wks of age, SjS mice show considerable manifestations of SjS-like disease evidenced by loss of secretory function, inflammatory infiltrates, and elevated autoAb levels [3]. At the study termination, histological analysis of the submandibular glands (SMGs) was performed on H&E-stained, paraffin-embedded sections (Fig. 2d). SMGs from low dose of LL-CFA/I-treated SjS females showed significant (*P* < 0.05) reduction in the number of inflammatory cell foci and foci area relative to those observed in PBS- and LL vector-treated mice (Fig. 2d, e). The foci area and number of foci were not significantly different between PBS- and LL vector-treated SMGs. SjS mice treated with the high-dose LL-CFA/I showed no significant reduction in the foci score nor foci area and were not significantly different for those from LL vector- or PBS-treated groups.

Contributing to SjS pathogenesis is the development of antinuclear antibodies (ANA) [18, 63]. Inquiring into whether CFA/I fimbriae can deter their production, ANA determinations were made in mice from PBS-, LL vector-, and LL-CFA/I-treated groups. Individual serum from SjS mice treated with the low-dose of LL-CFA/I exhibited significantly (*P* < 0.05) less speckled ANA than PBS- and LL vector-treated groups (Fig. 2f). Collectively, these data showed that dosing with 5 × 10^7^ CFUs of LL-CFA/I every 3 wks provides SFR maintenance in SjS mice. Hence, the subsequent studies continued to use the 5 × 10^7^ CFUs dose of LL-CFA/I or LL vector for treating SjS mice.

The maintenance of SFR in SjS mice implicates intervention by Tregs. Examination of total mesenteric lymph node (MLN) CD4^+^ T cells revealed no significant reduction in their numbers amongst the treatment groups (Fig. 2g). Despite relatively similar frequency of CD4^+^ T cells, LL-CFA/I low-dose treatment resulted in a 4- and 4.2-fold increase in the frequency of Foxp3^+^ CD4^+^ T cells relative to PBS- and LL vector-treated mice, respectively (Fig. 2h). The percentages of Foxp3^+^ CD4^+^ T cells represented 13% of all CD4^+^ T cells compared to 4% and 3% in PBS- and LL vector-treated mice, respectively. Upon probing further into whether the Tregs induced by LL-CFA/I expressed CD25 (Fig. 2i), results showed relatively fewer Foxp3^+^ CD25^+^CD4^+^ T cells by approximately one third of the Foxp3^+^ CD4^+^ T cells. Nonetheless, the percentages of Foxp3^+^ CD25^+^ CD4^+^ T cells were significantly (*P* < 0.05) increased ~ 3-fold by the low-dose LL-CFA/I-treated mice relative to PBS- and LL vector-treated mice (Fig. 2j). Although the frequency of CD25^+^CD4 T cells was also significantly (*P* < 0.05) elevated relative to PBS and LL vector treatment groups (Fig. 2i), the Foxp3^+^ CD25^+^ CD4^+^ T cells represented only a minor portion of Foxp3^+^ Tregs, and the majority remained as Foxp3^+^ CD25^−^ CD4^+^ T cells (Fig. 2h, j). Hence, LL-CFA/I treatment augments Treg induction, and these Tregs are heterogeneous in their expression of Foxp3 and CD25.

### Treatment with LL-CFA/I reduces IL-17 and IFN-γ

**Fig. 3:**
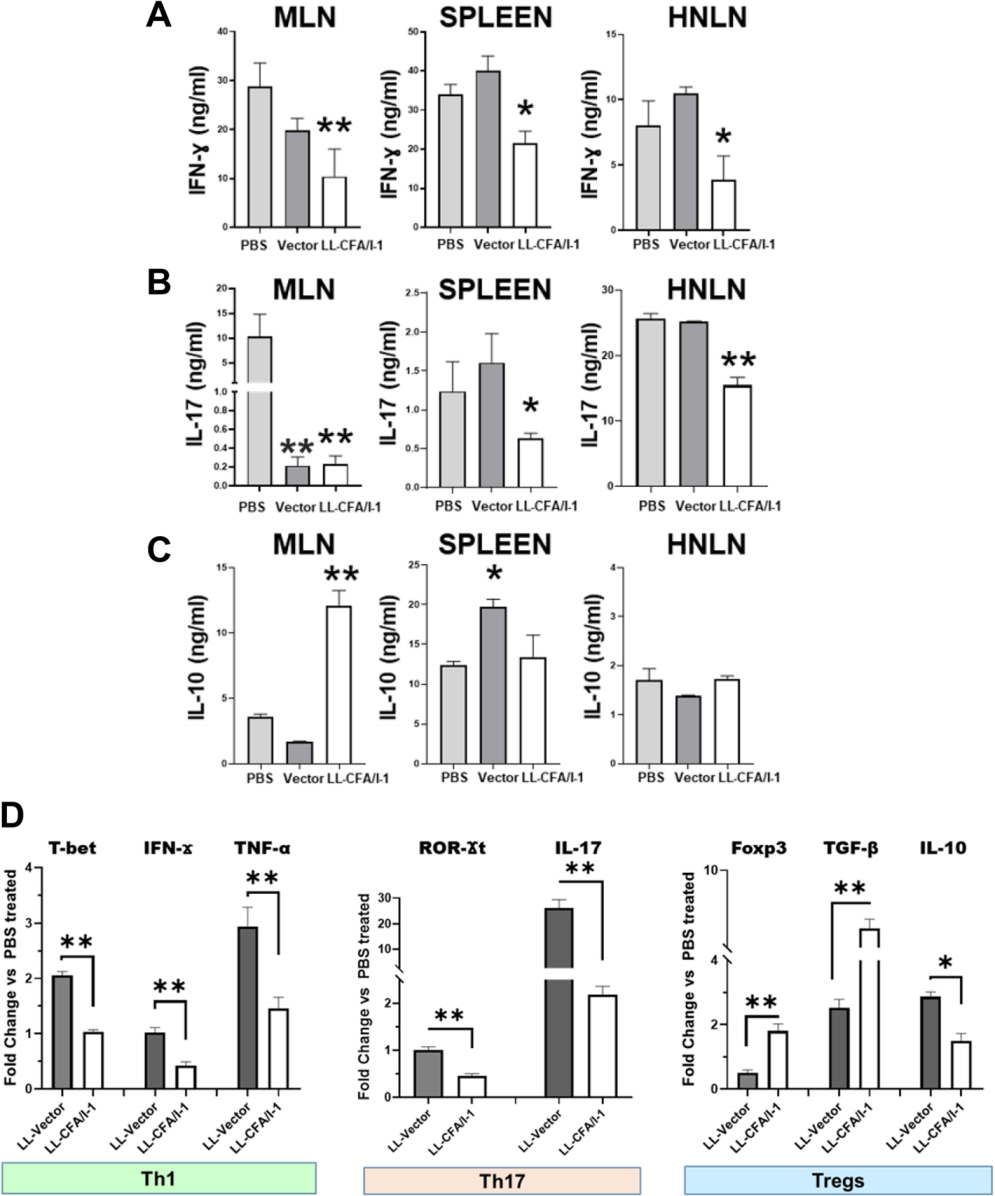
LL-CFA/I treatment augments IL-10 production and diminishes IFN-γ and IL-17. MLN, splenic, and HNLN lymphocytes were stimulated with anti-CD3 plus anti-CD28 monoclonal antibodies (mAbs) for 4 days. Collected culture supernatants were analyzed for production of **a** IFN-γ, **b** IL-17, and **c** IL-10. Depicted are the means ± SEM of duplicate cultures from individual mice; *P < 0.05, **P < 0.01 for LL-CFA/I versus LL vector or PBS groups. **d** mRNA analysis of Th1, Th17, and Treg cells subsets was conducted. RNA extracted from 2-day anti-CD3 plus anti-CD28 mAb-stimulated MLN lymphocytes from PBS-, medium dose LL vector-, and low-dose LL-CFA/I-treated groups (4 mice/group) were analyzed by QRT-PCR for **Th1**: T-bet, IFN-γ, and TNFα; **Th17**: Rorγt and IL-17; and **Tregs**: Foxp3, TGF-β, and IL-10 mRNA expression. Fold changes versus expression obtained by lymphocytes from the PBS group are depicted. Significance was determined using a unpaired *t* test for comparisons: *P < 0.05, **P < 0.01 LL CFA/I compared to LL vector-treated group

To determine whether LL-CFA/I reduces proinflammatory cytokine production, mononuclear cells from the MLNs, spleens, and HNLNs were isolated from each treatment group at 28 wks of age, corresponding to 1 week after the last administered treatment. Lymphocytes were stimulated with plate-bound anti-CD3 plus soluble anti-CD28 monoclonal Abs (mAbs). Culture supernatants were collected following 4 days of stimulation for soluble cytokine measurement (Fig. 3a–c). IFN-γ production was significantly reduced by groups treated with the low and medium doses of LL-CFA/I compared to PBS and LL vector treatment groups (Additional File 2 A and Fig. 3a). In a dose-dependent fashion by splenic and HNLN lymphocytes, LL-CFA/I significantly (*P* < 0.01) diminished IL-17 production relative to those groups treated with PBS or LL vector (Fig. 3b). Although MLN IL-17 was reduced by the low dose of LL-CFA/I, LL vector-treated group also showed IL-17 suppression, but the LL vector-mediated suppression was not evident in the spleens and HNLNs (Fig. 3b). The amount of anti-inflammatory cytokine, IL-10, was significantly (*P* < 0.01) increased by splenic and MLN lymphocytes from the LL-CFA/I-treated group compared to LL vector and PBS treatment groups (Fig. 3c). Splenic IL-10 was increased for the LL vector- and high-dose LL-CFA/I-treated groups (Additional File 2A). No significant difference in HNLN IL-10 production was observed amongst any of the treated groups, suggesting that low-dose LL-CFA/I treatment effectively suppresses systemic inflammation in SjS mice.

To investigate the types of Th cell subsets present in diseased and treated groups, cytokine and transcription factor mRNA analysis was performed on restimulated MLN lymphocytes. The level of cytokine mRNA expression for the low-dosed LL-CFA/I was compared to LL vector-treated group. For Th1 cells, T-bet transcription factor was significantly (*P* < 0.01) decreased for the LL-CFA/I-treated groups compared to LL vector. Also, LL-CFA/I treatment showed a reduced amount of IFN-γ and TNF-α mRNA compared to LL vector. (Fig. 3d). Treatment with LL-CFA/I group significantly (*P* < 0.01) reduced RORγt transcription factor and IL-17 gene expression compared to LL vector-treated group (Fig. 3d). Low-dose treatment with LL-CFA/I also increased the Treg transcriptional signature as Foxp3 and TGF-β were upregulated relative to LL vector-treated mice. IL-10 mRNA was also significantly (*P* < 0.05) increased in mice treated with LL vector relative to the low-dose LL-CFA/I treatment demonstrating that low-dose LL-CFA/I treatment suppresses pathogenic Th1 and Th17 cell responses potentially via IL-10-producing Tregs (Fig. 3d).

### LL-CFA/I treatment maintains SFR and diminishes proinflammatory cytokine production via stimulation of IL-10

**Fig. 4:**
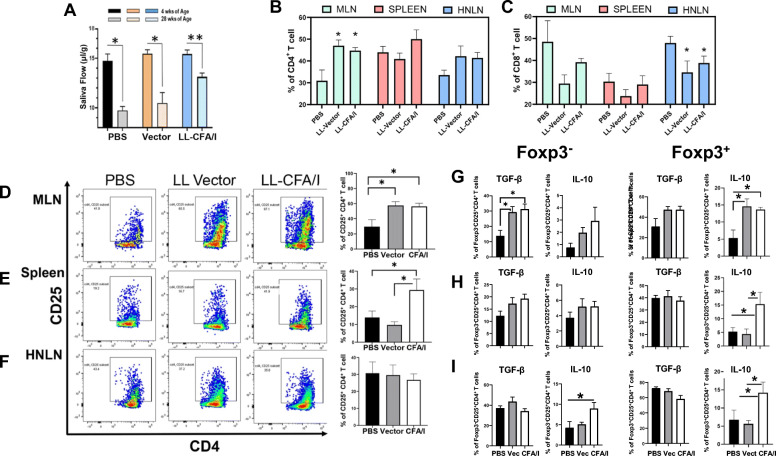
Treatment with *L. lactis*-CFA/I induces Tregs and sustains SFR in B6.NOD-*Aec1Aec2* (SjS) female mice. Ten wk-old SjS females (*n* = 7–8/group) were orally dosed with 5 × 10^7^ CFUs of LL-CFA/I, LL vector (Vec.), or PBS, and additional doses given every 4 wks. **a** SFR depicted for mice at 4 wks of age before treatments compared to treated mice at 28 wks of age. The PBS-treated group at termination showed a mean SFR of only 7.83 ± 0.83 μl/g. Significant changes in SFR from 4 wks measurement are depicted: *P < 0.05, **P < 0.01. **b**–**i** Two wks after their final dose, density gradient-purified lymphocytes from MLNs, spleens, and HNLNs were stimulated for 2 days with anti-CD3 and anti-CD28 mAbs. The percent MLN **b** CD4^+^ and **c** CD8^+^ T cells are shown for all treatment groups. **d**–**f** Representative FACS plots and histograms show the percent CD25^+^CD4^+^ T cells in the **d** MLNs, **e** spleens, and **f** HNLNs. Additional analysis was performed to discern the percentage of **g** MLN, **h** splenic, and **i** HNLN Foxp3^−^ and Foxp3^+^ CD25^+^ CD4^+^ T cells expressing TGF-β and IL-10; *P < 0.05, **P < 0.01 reflect differences from PBS-treated group

Studies were repeated to appraise the low-dose capacity of LL-CFA/I to minimize salivary flow loss. SjS females were dosed at 4-wk intervals throughout the study until termination at 28 wks of age (Fig. 4). SjS SFR was measured at week 4 prior to onset of treatment, and mice were dosed five times with 5 × 10^7^ CFUs of LL-CFA/I, LL vector, or PBS beginning at week 10. At week 28, only the LL-CFA/I-treated group showed significantly (*P* < 0.05) enhanced SFR versus LL vector- or PBS-treated SjS females, which showed no effect (Fig. 4a).

Upon termination of the study, T cell analysis was performed to examine the distribution of CD4^+^ and CD8^+^ T cells in the MLNs, spleen, and HNLNs (Fig. 4b, c). The percent CD4^+^ T cells increased significantly (*P* < 0.05) in the MLNs compared to naïve SjS females, but not in the spleen or HNLNs. No significant difference in the frequency of CD8^+^ T cells in the MLNs and spleens was observed; however, the percentage of CD8^+^ T cells in HNLNs was reduced in the LL vector- and LL-CFA/I-treated SjS females.

To assess what type of Tregs was induced in SjS mice following LL-CFA/I treatment, flow cytometry analysis was performed on lymphocytes isolated from the MLNs, spleens, and HNLNs from each treatment group and found that the frequency of CD25 expression in MLNs and spleen increased two-fold (*P* < 0.05) in mice treated with LL-CFA/I over those treated with PBS (Fig. 4d, e). Interestingly, the percentage of MLN CD25^+^CD4^+^ T cells also increased in LL vector-treated SjS females, showing that LL vector itself has an effect. The frequency of CD25^+^CD4^+^ T cells in the HNLNs remained unchanged by LL-CFA/I treatment (Fig. 4f). Further analysis of CD25^+^CD4^+^ T cells being Foxp3^+^ revealed that 6.9% were Foxp3^+^ (data not shown) similar to that obtained (7.1%) in Fig. 2j.

To determine the types of regulatory cytokines produced by the LL-CFA/I-induced Tregs, additional flow cytometry analysis revealed an increased frequency of IL-10^+^ Foxp3^+^ CD25^+^ CD4^+^ T cells (Tregs) in all tissues examined subsequent LL-CFA/I treatment (Fig. 4g–i). A portion of the Foxp3^+^ Tregs also expressed TGF-β, but there was no correlation with treatment (Fig. 4g–i). However, examination of Foxp3^−^ Tregs revealed significant increases (*P* < 0.05) in the frequency of MLN TGF-β^+^ Foxp3^−^ Tregs and HNLN IL-10^+^ Foxp3^−^ Tregs (Fig. 4g–i). For the remaining tissues, no significant difference in IL-10 expression was observed. Thus, Tregs were heterogeneous in their expression of Foxp3 and regulatory cytokines.

**Fig. 5:**
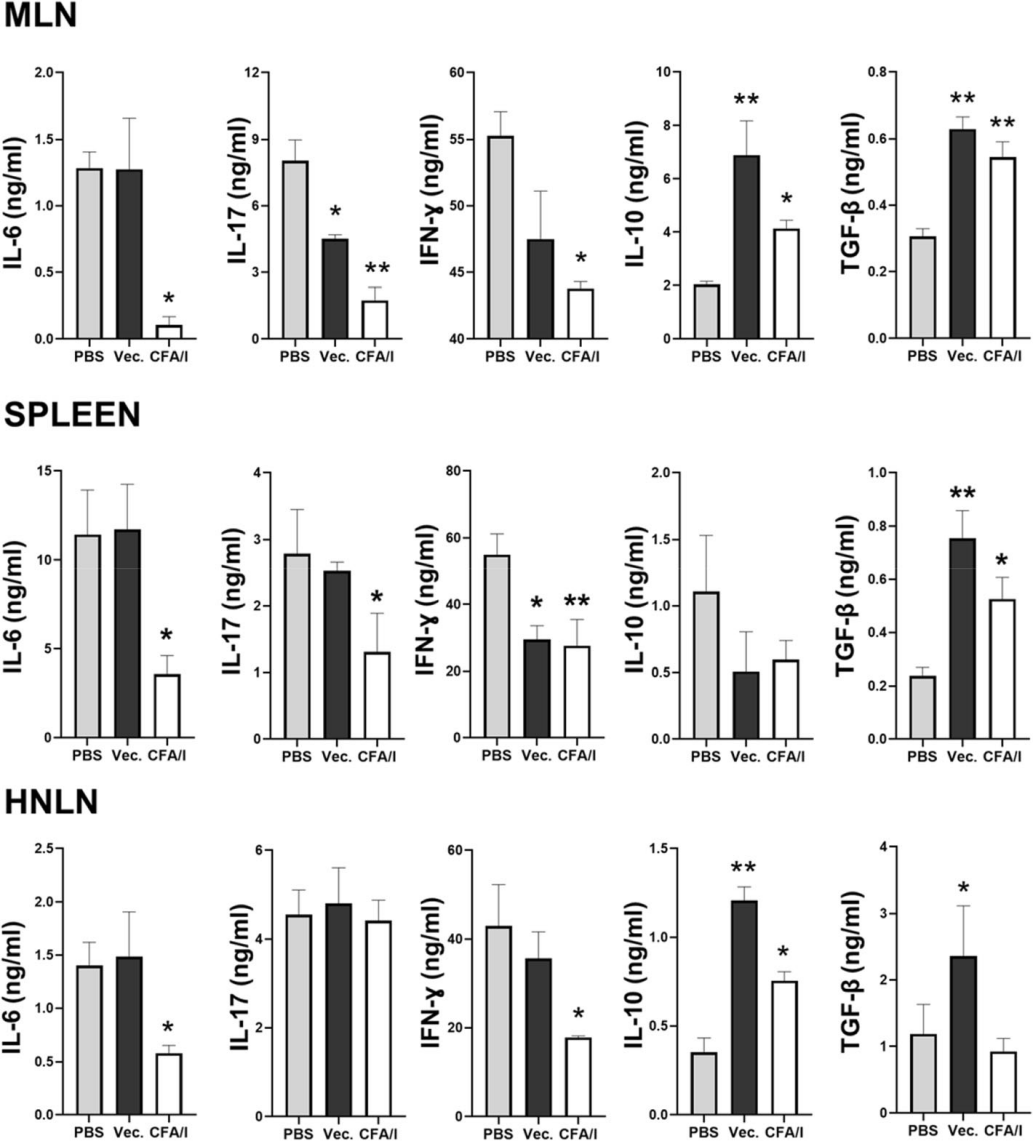
Treatment with *L. lactis*-CFA/I suppresses proinflammatory cytokines and augments regulatory cytokine production. Lymphocytes from **a** MLNs, **b** spleens, and **c** HNLNs were isolated from treatment groups described in Fig. 4. At study termination, purified lymphocytes from PBS-, LL vector (Vec.)-, and LL-CFA/I-treated mice were stimulated with anti-CD3 and anti-CD28 mAbs for 4 days for cytokine ELISA. Culture supernatants were analyzed for production of IL-6, IL-17, IFN-γ, IL-10, and TGF-β by cytokine-specific ELISAs. Depicted are the means ± SEM; *P < 0.05 and **P < 0.01 versus PBS-dosed mice

To assess to the degree that LL-CFA/I can suppress proinflammatory cytokine production, total T cells were stimulated with anti-CD3 plus and anti-CD28 mAbs for 4 days, and cytokine-specific ELISAs were performed on culture supernatants. IL-6 was significantly suppressed 3- to 12-fold by LL-CFA/I treatment in the MLNs, spleens, and HNLNs when compared to PBS-treated mice (Fig. 5a–c. LL-CFA/I also significantly suppressed IL-17 3- to 5-fold, and IFN-γ by 1.2- to 2.2-fold (Fig. 5a–c). MLN IL-17 levels were reduced by LL vector treatment, but not for splenic and HNLN T cells. In contrast, IL-10 was significantly (*P* < 0.05) increased by LL-CFA/I treatment compared to PBS-treated mice in MLNs and HNLNs by 2-fold (Fig. 5a, c). TGF-β was significantly (*P* < 0.05) increased by LL-CFA/I treatment in MLNs and spleen by 1.7 and 2.5-fold, respectively (Fig. 5a, b). Hence, LL-CFA/I is anti-inflammatory evidenced by increased IL-10 and TGF-β from CD4^+^ T cells and reduced production of proinflammatory cytokines.

### Adoptive transfer of CD4^+^ T cells from LL-CFA/I-treated donors induced Tregs and reduced IL-17- and IFN-γ-producing CD4^+^ T cells in diseased SjS recipients

**Fig. 6:**
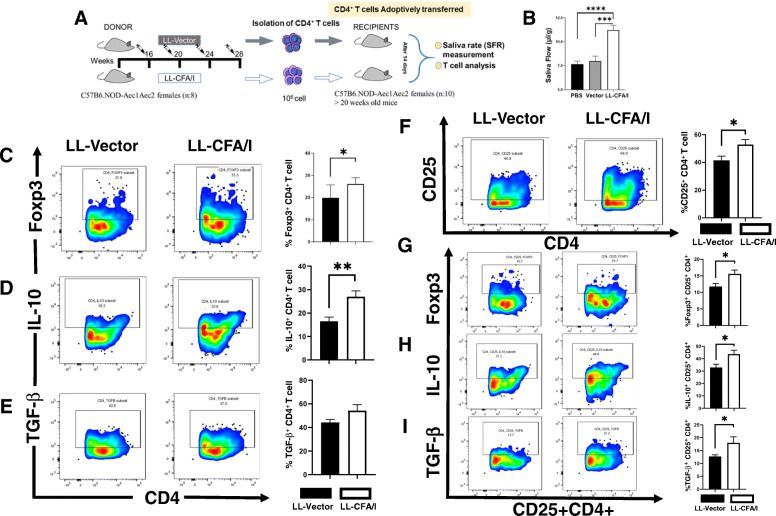
Adoptive transfer of total CD4^+^ T cells from LL-CFA/I-treated SjS mice restores SFR and increases both the frequency and activity of Tregs in recipient diseased SjS mice. **a** The donor pool composed of SjS females (8/group; 16 wks of age) treated four times at 4 wk intervals with either LL vector or LL-CFA/I similar to that described above. Two wks following the last dose given on wk 28, total LN and splenic CD4^+^ T cells were purified to > 95% purity, and 1 × 10^6^ CD4^+^ T cells/mouse were i.v. injected into recipient SjS females (> 20 wks of age) (10/group). The percentage of CD25^+^CD4^+^ T cells in each group was measured 2 wks later. **b** Only recipients given CD4^+^ T cells from LL-CFA/I-treated mice showed restored SFR; ***P < 0.0001, ****P < 0.00001 versus LL vector-treated mice. MLN Treg composition as percent CD4^+^ T cells in the recipients are shown in representative FACS plots and histograms: **c** Foxp3^+^, **d** IL-10^+^, and **e** TGF-β^+^. **f**–**h** Frequency of CD25^+^CD4^+^ T cell subsets as **f** CD25^+^CD4^+^, **g** Foxp3^+^ CD25^+^CD4^+^, **h** IL-10^+^ CD25^+^CD4^+^, and **i** TGF-β^+^ CD25^+^CD4^+^ T cells in recipients are depicted; *P < 0.05, **P < 0.01 for comparison between recipients given donor CD4^+^ T cells from LL-CFA/I or LL vector. Data are representative of two experiments

In an effort to understand how CD4^+^ T cells mediate their protection against SjS, CD4^+^ T cells were purified from LNs and spleens of LL vector- or LL-CFA/I-treated SjS donors. Donor SjS females at 16 wks of age were orally treated four times at 4-wk intervals (Fig. 6a). Two weeks after the last dose, CD4^+^ T cells were purified from LL vector- or LL-CFA/I-treated mice. Pooled CD4^+^ T cells from each group were intravenously injected with 1 × 10^6^ CD4^+^ T cells/mouse into naïve 20 wk-old SjS females showing reduced SFR. After 14 days, SFR was analyzed, and only the recipients given CD4^+^ T cells from LL-CFA/I-treated donors showed significant (*P* < 0.0001) restoration of salivary flow (Fig. 6b). Neither PBS nor LL vector recipients showed any restoration of salivary flow (Fig. 6b).

**Fig. 7:**
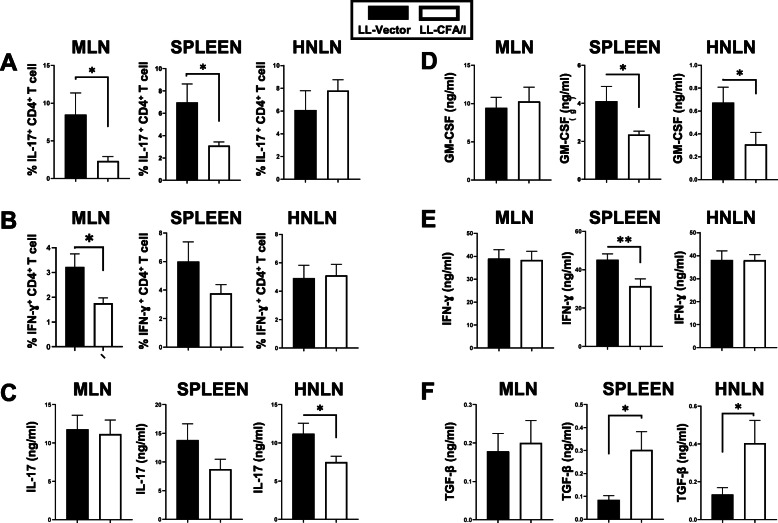
CD4^+^ T cell SjS recipients from LL-CFA/I-treated donors showed reduced proinflammatory cytokine production and increased TGF-β production. Recipient lymphocytes isolated from the MLNs, spleens, and HNLNs were restimulated with anti-CD3 plus anti-CD28 mAbs. **a**, **b** After 2 days, lymphocytes were harvested for flow cytometry analysis to measure percent **a** IL-17^+^ and **b** IFN-γ^+^ CD4^+^ T cells. Collected cell culture supernatants from 4-day restimulated cultures were analyzed for the presence of **c** IL-17, **d** GM-CSF, **e** IFN-γ, and **f** TGF-β. *P < 0.05, **P < 0.01, differences in recipients given donor CD4^+^ T cells from LL vector- or LL-CFA/I-treated SjS mice are shown

Examination of the Treg composition in the recipients revealed a significant increase in the frequency of MLN Foxp3^+^ CD4^+^ and CD25^+^ CD4^+^ T cells, respectively (Fig. 6c, f). However, only 15% of the CD25^+^ CD4^+^ T cells expressed Foxp3 (Fig. 6g), unlike the percent Foxp3^+^ CD4^+^ T cells showed a greater percentage of ~ 28% (Fig. 6c). An increase in the frequency of IL-10^+^ CD4^+^ T cells was also noted (Fig. 6d), as well as, an increase in frequency of IL-10 and TGF-β associated with CD25^+^ CD4^+^ T cells (Fig. 6h, i). Furthermore, the frequency of IL-17- and IFN-γ-producing CD4^+^ T cells was determined. Recipients given LL-CFA/I-induced Tregs showed significantly fewer IL-17- and IFN-γ-producing CD4^+^ T cells than recipients given CD4^+^ T cells from LL vector-treated SjS mice (Fig. 7a, b). Analysis of culture supernatants by cytokine ELISA revealed that GM-CSF and IFN-γ are reduced 2-fold in the spleen (Fig. 7d, e), and IL-17 is reduced 2-fold in the HNLNs (Fig. 7c). In contrast, TGF-β is significantly (*P* < 0.05) enhanced in the spleens and HNLNs (Fig. 7f). IL-10 showed no difference in LL-CFAI recipient’s tissues compared to LL vector recipients (data not shown). Thus, adoptive transfer of regulatory cells contained within the CD4^+^ T cell population is able to confer protection against further progression of SjS symptoms.

## Discussion

SjS is a chronic, progressive autoimmune disease responsible for severe dryness of the eyes and mouth. Inflammation in the exocrine glands results in reduced secretion of tears and saliva [64, 65]. Few studies have addressed the etiology of this disorder; thus, current interventions only treat the symptoms and do not impact the destructive autoimmune process underlying SjS. The development of effective therapeutics for SjS poses significant challenges, in part because SjS is often associated with other autoimmune disorders, e.g., lupus and arthritis.

The C57BL/6.NOD-*Aec1Aec2* mice are a well-described SjS mouse model showing an early onset of sialadenitis in females with an initial high T cell infiltration followed by an increased B cell infiltration [19]. To address whether an immune intervention strategy could attenuate SjS development, oral treatments with CFA/I fimbriae were tested for their therapeutic potential as has been done for other autoimmune disease models including multiple sclerosis [32], arthritis [34, 37], and type 1 diabetes [35]. The conferred protection to these diseases is attributed to an increase in Tregs in an auto-Ag-independent fashion. CFA/I fimbriae possess anti-inflammatory properties capable of further reducing the development of autoimmune disease. Using the SjS mouse model, a prophylactic strategy was tested to inhibit the onset and development of SjS disease noted by the stimulation of regulatory cytokines, IL-10 and TGF-β. Treatment with LL-CFA/I, not LL vector, offers a means to ameliorate SjS by suppressing proinflammatory cytokines that reduce both the innate and adaptive immune responses.

A striking feature of LL-CFA/I treatment in SjS females is that salivary flow was preserved, but not with the LL vector or PBS treatments. The preserved salivary flow was attributed to the significant reduction in the number of inflammatory foci and size of lesions as a consequence of LL-CFA/I treatment. Although different doses of LL-CFA/I were tested, optimal results were achieved with as little as 5 × 10^7^ CFUs administered at 3- or 4-week intervals. Why the lack of SFR maintenance was not obtained with the high dose of LL-CFA/I is unclear, but a similar effect was observed when treating NOD mice with type 1 diabetes [35]. Possibly, the overstimulation of the host ligand for CFA/I fimbriae may shut down the intended response. Studies to assess the natural host ligands for CFA/I fimbriae are ongoing. The concomitant reduction in the number of foci and foci area seen in the SMGs provided evidence that LL-CFA/I treatment protects against inflammation of these glands. Past studies have shown the influence of Th17 cells driving the development of SjS in this animal model, resulting in increased pathogenic speckled autoAb profiles [22, 25, 26, 32]. The potent activation of autoreactive B and T cells, which was found to be highly upregulated at the clinical-disease stage, is known to contribute to rapid loss of salivary gland function in female SjS mice [19]. Notably, the ANA levels were significantly reduced as a consequence of LL-CFA/I treatment.

Proinflammatory cytokine production has been found to correlate to exocrine gland dysfunction [66–69]. Th17/Treg cell imbalance leads to the breakage of immunological tolerance, and the loss or reduced function of Tregs can have pathological consequences enabling the development of various autoimmune diseases [70]. In fact, SjS patients show an increased presence of Th17 cells in their salivary glands [22, 23, 68]. Examination of Th17-type cytokines revealed that LL-CFA/I treatment significantly reduced IL-6, GM-CSF, and IL-17 production by all lymphoid tissues examined. The mRNA for the Th17 transcription factor, RORγt, was also significantly reduced, implicating the reduction in IL-17 activation. IFN-γ is a key cytokine involved in a Th1 cell response, and overexpression of IFN-γ and elevated Th1-type responses can contribute to autoimmune pathogenesis, such as rheumatoid arthritis and multiple sclerosis [71]. IFN-γ has been shown to contribute to SjS pathogenesis in humans [71] and in the SjS mice [25]. Low-dose LL-CFA/I treatment was found to suppress IFN-γ production in all tissues examined, as well as, reducing the mRNA for the Th1 cell transcription factor, T-bet. Hence, oral treatment with LL-CFA/I suppresses the development of proinflammatory cytokines.

LL-CFA/I treatment showed a marked enhancement in Treg levels, which is a notable finding for the treatment of SjS. Most studies in the past have focused on the characterization of effector T cells responsible for sustaining disease. The reduced proinflammatory cytokine production correlated to increased IL-10 and TGF-β production by lymphoid tissues in LL-CFA/I-treated mice, although the specific amount and source varied. A pan-Treg marker was not evident for the SjS studies as with past CIA studies, whereby all Tregs were contained within the CD39^+^ CD4^+^ T cell subset [34, 37]. Upon examination of CD39 expression, results showed low amount of expression compared to CD25^+^ CD4^+^ T cells and excluded from a portion of the Foxp3^+^ Tregs (data not shown). Underscoring their relevance for treating SjS, the current study offers viable alternatives for treating SjS by stimulating Tregs and other anti-inflammatory cells. The data showed at least two methods of inflammatory cell suppression. First, cytokine analysis of whole lymphocyte cultures from all tissues examined revealed suppression of IL-6, IL-17, and IFN-γ. Such results are indicative of regulatory cell suppression, via Tregs or other regulatory cell subsets. Subsequent to adoptive transfer of LL-CFA/I-derived CD4^+^ T cells netted reductions in Th17 cells noted by their specific reductions in frequencies of IL-17^+^ CD4^+^ T cells in the MLNs and spleens. Furthermore, soluble cytokine quantification revealed varied reductions in IL-17-, GM-CSF-, and IFN-γ-producing cells from MLNs, spleens, and HNLNs. Such varied production may be linked to the ability of the donor CD4^+^ T cells to migrate to the various tissues and/or may require additional antigen-presenting cells (APCs) to sustain long-lived Tregs. The impact of these donor CD4^+^ T cells may be short-lived if these are not sustained in the different lymphoid compartments. Nevertheless, from the adoptive transfer studies, transient disease suppression by Tregs is one mechanism of action by LL-CFA/I, suggesting active Treg suppression occurs when LL-CFA/I is directly used to treat disease. In addition to the reduced frequency of Th17 and Th1 cells, increases in anti-inflammatory cytokine production revealed varied production of IL-10 and TGF-β by the MLNs, spleens, and HNLNs. Although differences were observed amongst these tissues, either IL-10 or TGF-β was increased by LL-CFA/I, but one or both cytokines are often necessary to regulate inflammation and subdue autoimmunity. Why such variations occur remains unclear, but current studies are examining Treg diversity in more detail to determine if the route of therapeutic treatment is important. Route of delivery may be why TGF-β levels were significantly increased only in the spleens and HNLNs and not in the MLNs, given that the donor CD4^+^ T cells were delivered systemically.

Secondly, evaluation of elicited Tregs was found to contain both CD25^+^ and CD25^−^ and/or Foxp3^+^ and Foxp3^−^. Similar Treg diversity was also observed when LL-CFA/I was used to prevent type 1 diabetes development [35], and varied Treg subsets induced with CFA/I fimbriae also were able to prevent and/or diminish arthritis [36, 37]. In fact, Foxp3^−^ Tregs interchanged with Foxp3^+^ Tregs and vice-versa subsequent adoptive transfer [36]. In the current studies, such variability amongst the different tissue examined also occurred. LL-CFA/I clearly exhibited suppressive capacity on SjS noted by the preserved salivary flow, whereas PBS or LL vector treatment did not. Instead, the evidence demonstrates Foxp3 associating with both CD25^−^ and CD25^+^ CD4^+^ T cell subsets. Analysis in this study revealed that although TGF-β associated with both Foxp3^−^ and Foxp3^+^ CD4^+^ T cell subsets, IL-10 segregated mostly with Foxp3^+^ CD4^+^ T cells similar to what we found with past studies using CFA/I fimbriae [32–36]. Treg responses are offered as one possible mechanism. In some instances, Tregs were difficult to distinguish solely by their phenotype, resulting in presumed Treg levels not being significantly different from levels in PBS- nor LL vector-treated mice. As depicted in Fig. 4, Tregs were not phenotypically different in the MLNs from the LL vector-treated mice nor in the HNLNs from any of the treated groups. Although LL vector did show increases in MLN and HNLN CD25^+^ CD4^+^ T cells, its impact is mostly local and did not translate into Tregs. Since CD25 is also an indicator of activation, perhaps a portion of the elicited CD25^+^ CD4^+^ T cells is effector T cells. Further inquiry into their cytokine profiles revealed evidence for Tregs being more pronounced with those elicited by LL-CFA/I treatment. IL-10 did positively associate with Foxp3^+^ CD25^+^ Tregs in all tissues examined, but neither LL vector nor PBS treatment could elicit IL-10^+^ Foxp3^+^ CD25^+^ Tregs in distal spleen and HNLNs. LL vector may have some regulatory inducing capabilities locally as suggested by the increased Foxp3^+^ CD25^+^ CD4^+^ T cells in the MLNs. In contrast, TGF-β did associate with both Foxp3^+^ CD25^+^ and Foxp3^−^ CD25^+^ Tregs in MLNs, spleens, and HNLNs. Often, baseline anti-inflammatory cytokines are induced to regulate inflammatory cytokine responses, and this may account for what was observed for TGF-β. Importantly, the frequency of IL-17- and IFN-γ-producing CD4^+^ T cells was reduced subsequent to LL-CFA/I treatment. Hence, these studies demonstrate restoration of salivary flow via the protective capacity of CFA/I fimbriae to diminish inflammatory cytokines by the stimulation of anti-inflammatory cytokines with the aid of induced Tregs.

## Conclusions

Indeed, these findings point to the capacity of CFA/I fimbriae to elicit bystander suppression via different Treg subsets uniquely equipped to suppress inflammation. These Tregs are heterogeneous in their expression of Foxp3, IL-10, and TGF-β and work collectively to prevent loss of salivary flow.

## Supplementary Information

**Additional file 1.** Specific growth conditions for LL-CFA/I and LL vector.

**Additional file 2.** LL-CFA/I treatment augments IL-10 production and diminishes IFN-γ and IL-17.

## Data Availability

All data generated or analyzed during this study are included in this article.

## Abbreviations

SjS: Sjögren’s syndrome
CFA/I: Colonization factor antigen I
Ag: Antigen
LL: *Lactococcus lactis*
PBS: Phosphate-buffered saline
SLE: Systemic Lupus Erythematosus
RA: Rheumatoid arthritis
NOD: Nonobese diabetic
autoAb: Autoantibody
M3R: Muscarinic receptor type 3
*lpr*: Lymphoproliferation gene
IFN: Interferon
NK: Natural killer
mAb: Monoclonal antibody
Th: T helper
Tregs: Regulatory T cells
EAE: Experimental autoimmune encephalomyelitis
LAB: Lactic acid bacteria
B6: C57BL/6
CFUs: Colony-forming units
SFR: Salivary flow rate
IP: Intraperitoneal
H&E: Hematoxylin and eosin
HNLNs: Head and neck lymph nodes
MLNs: Mesenteric LNs
SMGs: Submandibular glands
