# Supplementary material for: Stimulation of regulatory T cells with Lactococcus lactis expressing enterotoxigenic E. coli colonization factor antigen 1 retains salivary flow in a genetic model of Sjögren’s syndrome
Source: Arthritis Res Ther. 2021 Apr 6;23:99. doi: 10.1186/s13075-021-02475-1 (PMC8022426; doi:10.1186/s13075-021-02475-1)
Supplement: Supplementary file 1 — Additional file 1. Specific growth conditions for LL-CFA/I and LL vector. [file 13075_2021_2475_MOESM1_ESM.docx]

**Additional File 1**

**Specific growth conditions for LL-CFA/I and LL vector**

*L. lactis* IL1403 cultures were induced exactly at OD_600_=0.2, and the plasmid, pMSP3535H3, was previously developed [67]. The strains were inoculated in 6 ml of M17 0.5% glucose for overnight pre-culture at 32^o^C. The following morning, 3 ml of the preculture were transferred splash-less into 50 ml of the same pre-warmed medium, and grown to the required OD, normally taking 1.5 - 2 hrs. The utmost care was taken to avoid any mechanical disturbance of the culture, including vibrations. To mix nisin into the medium, brief swirling was applied. We empirically determined the best recombinant protein production was achieved for each strain to be 4 hours.
