# Supplementary material for: Stimulation of regulatory T cells with Lactococcus lactis expressing enterotoxigenic E. coli colonization factor antigen 1 retains salivary flow in a genetic model of Sjögren’s syndrome
Source: Arthritis Res Ther. 2021 Apr 6;23:99. doi: 10.1186/s13075-021-02475-1 (PMC8022426; doi:10.1186/s13075-021-02475-1)
Supplement: Supplementary file 2 — Additional file 2. LL-CFA/I treatment augments IL-10 production and diminishes IFN-γ and IL-17. [file 13075_2021_2475_MOESM2_ESM.docx]

**Additional File 2: LL-CFA/I treatment augments IL-10 production and diminishes IFN-γ and IL-17.**

**Additional File 2:**

Splenic, MLN, and HNLN lymphocytes were stimulated with anti-CD3 plus anti-CD28 monoclonal antibodies (mAbs) for 4 days. Collected culture supernatants were analyzed for production of (A) IFN-γ, IL-17, and IL-10. Depicted are the means ± SEM of duplicate cultures from individual mice; *P < 0.05, **P < 0.01 for LL-CFA/I versus LL vector or PBS groups. (B) mRNA analysis of Th1, Th17, and Treg cells subsets was conducted. RNA extracted from 2-day anti-CD3 plus anti-CD28 mAb-stimulated MLN lymphocytes from PBS-, medium dose LL vector (Vec.) and LL-CFA/I-treated groups (4 mice/group) were analyzed by QRT-PCR for **Th1**: T-bet, IFN-γ and TNFα; **Th17**: Rorγt and IL-17; and **Tregs**: Foxp3, TGF-β, and IL-10 mRNA expression. Fold changes versus expression obtained by lymphocytes from the PBS group are depicted. Significance was determined using a one-way ANOVA test followed by Tukey post-correction test for multiple comparisons: *P < 0.05, **P < 0.01 compared to LL vector-treated group.
